# Aqueous Extract of Saposhnikoviae Radix Antagonizes Arsenic Neurotoxicity via the NLRP3/Caspase-1/GSDMD Pyroptotic Axis: Translating Environmental Exposure into Neuropathology and Herbal Recalibration

**DOI:** 10.3390/toxics14080706

**Published:** 2026-08-10

**Authors:** Xiao-Ping Zeng, Yu Liu, Bin He, Ji-Gang Pan

**Affiliations:** 1 Key Laboratory for Basic and Clinical Research of Cardiovascular Diseases, Department of Physiology and Pathophysiology, School of Basic Medical Sciences, Guizhou Medical University, Guian New Area, Guiyang 561113, China; zengxiaoping@gmc.edu.cn (X.-P.Z.); m18385018851_2@163.com (Y.L.); 2 State Key Laboratory of Discovery and Utilization of Functional Components in Traditional Chinese Medicine, Engineering Research Center for the Development and Application of Ethnic Medicine and TCM (Ministry of Education), Guizhou Provincial Engineering Research Center for the Development and Application of Ethnic Medicine and TCM, School of Pharmacy, Guizhou Medical University, Guian New Area, Guiyang 561113, China; 3 Natural Products Research Center of Guizhou Province, Guiyang 550014, China

**Keywords:** neuroexposome, cognitive impairment, anxiety-like behavior, neuroinflammation, environmental neurotoxicant, traditional Chinese medicine

## Abstract

Chronic arsenic exposure causes neurological damage, but the underlying mechanisms remain incompletely understood. This study investigated whether Saposhnikoviae Radix (SR) aqueous extract protects against arsenic-induced neurotoxicity by modulating pyroptosis. Kunming mice were exposed to NaAsO_2_ (10 mg/kg) with or without SR (3, 6, 12 g/kg). NaAsO_2_ exposure induced anxiety-like behaviors and spatial memory deficits, accompanied by widespread neuronal damage (hippocampal pyramidal cell disarray, cerebellar Purkinje cell loss, and cortical vacuolation) and upregulation of pyroptosis-related proteins (NLRP3, Cleaved-Caspase-1, GSDMD-N, IL-1β, IL-18) in brain tissues. SR treatment significantly ameliorated these behavioral and pathological changes. In HT22 cells, NaAsO_2_ (12.5 μM) reduced cell viability, increased LDH release, induced pyroptotic ultrastructural features (membrane blebbing and pore formation), and upregulated pyroptosis markers; SR (400 μg/mL) effectively reversed these effects. Mechanistically, SR suppressed the NLRP3/Caspase-1/GSDMD axis activation, as confirmed by both protein and mRNA analyses. These findings demonstrate that SR aqueous extract attenuates arsenic-induced central nervous system injury through specific inhibition of the pyroptosis pathway, providing an experimental basis for the potential use of traditional Chinese medicine in preventing environmental metalloid-induced neurotoxicity.

## 1. Introduction

Arsenic, a ubiquitously distributed metalloid in groundwater and soil, represents a major environmental stressor within the emerging framework of the exposome. Chronic low-level exposure to arsenic via drinking water has been recognized as a global public health burden, with mounting evidence linking it to multi-organ toxicity affecting the skin, liver, cardiovascular system, and, notably, the central nervous system [1,2,3,4,5,6]. Within the neuroexposome paradigm—which seeks to integrate environmental exposures with physiological responses to understand brain health and disease—arsenic serves as a compelling case study, as it readily crosses the blood–brain barrier, accumulates in neural tissue, and induces a cascade of neurotoxic insults, including oxidative stress, mitochondrial dysfunction, endoplasmic reticulum stress, and neuronal apoptosis [7,8,9,10,11]. Epidemiologically and experimentally, chronic arsenic accumulation has been consistently associated with cognitive decline, memory impairment, anxiety, and depression-like neurobehavioral abnormalities, underscoring the urgent need to decipher the molecular bridges connecting this environmental exposure to neurological pathology [2,8,9,10,11,12,13,14,15,16].

Pyroptosis, a lytic and pro-inflammatory form of programmed cell death orchestrated by gasdermin family proteins, has emerged as a critical node linking environmental insults to neuroinflammation and tissue damage. Morphologically defined by cell swelling, membrane pore formation, and the concomitant release of mature IL-1β and IL-18, pyroptosis represents a fundamental pathway through which exogenous stressors perturb neuronal homeostasis [17,18,19,20,21,22]. The canonical pyroptotic cascade is initiated by the NLRP3 inflammasome: upon sensing diverse danger signals, NLRP3 oligomerizes with the Apoptosis-associated Speck-like protein containing a CARD (ASC) and pro-caspase-1 to form the inflammasome complex, which catalyzes the autocleavage of pro-caspase-1 into its active p20 fragment [23,24,25,26]. Active caspase-1 then executes a bifurcated downstream program: on one hand, it cleaves GSDMD to liberate the N-terminal fragment GSDMD-NT, which oligomerizes on the plasma membrane to form non-selective pores, causing osmotic imbalance and eventual membrane rupture [27,28]; on the other hand, it processes the inactive precursors pro-IL-1β and pro-IL-18 into their mature secretory forms, which are released through the membrane pores to amplify neuroinflammatory signals [29,30]. Importantly, arsenic exposure has been shown to activate the NLRP3 inflammasome and upregulate IL-18 and IL-1β in various models [24,28,31,32]. However, whether arsenic directly engages the GSDMD-dependent canonical pyroptosis pathway to drive neurotoxicity—and whether pharmacological blockade of this pathway can mitigate arsenic-induced neural injury—remains relatively unexplored, representing a critical gap in our understanding of the neuroexposome [33,34,35].

Saposhnikoviae Radix (SR), a perennial herb of the Apiaceae family, has a long history in traditional Chinese medicine, and interestingly, its aqueous extract has been documented for its efficacy in alleviating arsenic toxicity [36,37]. In recent years, accumulating pharmacological evidence has substantiated its neuroprotective potential: SR exerts stable beneficial effects in both cellular and animal models of Alzheimer’s disease and Parkinson’s disease by suppressing neuroinflammation, oxidative stress, and neuronal apoptosis [36,38,39,40,41,42,43,44]. Moreover, recent studies have demonstrated that SR, particularly its active component cimifugin, significantly inhibits NLRP3 inflammasome assembly and activation, thereby curtailing pyroptosis [45,46]. Nevertheless, the regulatory effect of SR on arsenic-elicited neuronal pyroptosis has not been investigated.

Collectively, these observations led us to formulate the hypothesis that SR aqueous extract mitigates sodium arsenite-induced neurotoxicity by suppressing NLRP3/Caspase-1/GSDMD axis-mediated pyroptosis. To test this hypothesis, we employed a NaAsO_2_-exposed mouse model and the HT22 mouse hippocampal neuronal cell line, systematically evaluating the neuroprotective efficacy of SR aqueous extract and its modulatory impact on the pyroptotic cascade across multiple biological scales—from whole-animal behavior and neuropathology to cytotoxicity, pyroptotic ultrastructure, and molecular expression signatures of core pathway components. Our findings aim to position arsenic neurotoxicity within the neuroexposome framework and identify SR as a potential pharmacological strategy that recalibrates exposure-driven neurophysiological dysregulation.

## 2. Materials and Methods

### 2.1. Ethics Statement

All animal experimental protocols were approved by the Experimental Animal Ethics Committee of Guizhou Medical University (Approval No. 2200304).

### 2.2. Experimental Materials and Reagents

*Saposhnikoviae Radix* (Batch No. 180214) was purchased from Beijing Tongrentang Pharmaceutical Co., Ltd. (Beijing, China). Botanical identification was performed by Professor Shaohuan Liu (School of Pharmacy, Guizhou Medical University) through macroscopic and microscopic characterization. A voucher specimen (No. 2021110701) has been deposited in the Herbarium of the School of Basic Medicine, Guizhou Medical University. Sodium arsenite (NaAsO_2_, CAS: 7784-46-5, purity ≥ 90%) was obtained from Sigma-Aldrich (St. Louis, MO, USA). Low-glucose DMEM was purchased from Gibco (Thermo Fisher Scientific, Waltham, MA, USA). Fetal bovine serum (FBS) was obtained from Biological Industries (Kibbutz Beit-Haemek, Israel). Penicillin/streptomycin was purchased from Thermo Fisher Scientific (Waltham, MA, USA). Phosphate-buffered saline (PBS) was purchased from Servicebio (Wuhan, China).

CCK-8 kit was purchased from Nanjing Jiancheng Bioengineering Institute (Nanjing, China). Lactate dehydrogenase (LDH) release assay kit (Cat. No. A020-2) was also obtained from Nanjing Jiancheng. Hematoxylin and eosin (H&E) staining kit, radioimmunoprecipitation assay (RIPA) lysis buffer, and phenylmethylsulfonyl fluoride (PMSF) and 5× SDS-PAGE loading buffer were purchased from Solarbio Science & Technology Co., Ltd. (Beijing, China). PVDF membranes (0.22 μm, Cat. No. ISEQ00010) were purchased from Millipore (Burlington, MA, USA). ECL chemiluminescent substrate (Cat. No. WBKLS0500) was purchased from Millipore (Burlington, MA, USA). TRIzol reagent, cDNA reverse transcription kit, and fluorescence quantitative PCR kit were purchased from Biomed (Beijing, China).

Primary antibodies used were as follows: NLRP3 (Cat. No. AF2155, Beyotime, Shanghai, China), Caspase-1 (Cat. No. 22915-1-AP, Proteintech, Wuhan, China), GSDMD (Cat. No. 39754, CST, Danvers, MA, USA), GSDMD-N (Cat. No. ab215203, Abcam, Cambridge, UK), IL-1β (Cat. No. BS6067, Bioworld Technology, Nanjing, China), IL-18 (Cat. No. AF5207, Beyotime, Shanghai, China), and β-actin (Cat. No. 66009-1-Ig, Proteintech, Wuhan, China). HRP-conjugated anti-rabbit IgG secondary antibody (Cat. No. BS13278, Bioworld Technology, Nanjing, China) was used for detection.

### 2.3. Preparation of Saposhnikoviae Radix Aqueous Extract

The *Saposhnikoviae Radix* (SR) used in this study was derived from the dried root slices of *Saposhnikovia divaricata* (Turcz.) Schischk. The medicinal material was authenticated by Professor Shaohuan Liu from the School of Pharmacy, Guizhou Medical University, and a voucher specimen (No. 2021110701) was deposited in the Physiology Laboratory, Rooms 5–9, School of Basic Medicine, Guizhou Medical University. According to a previously reported standardized water decoction extraction protocol [37] for SR, two types of stock solutions were prepared for in vitro cell intervention and in vivo mouse gavage, respectively.

#### 2.3.1. Preparation of Stock Solution for Cell Experiments

Accurately weigh 10 g of dried SR, add 800 mL of double-distilled water, and decoct for 30 min. Repeat the decoction three times. Combine the three filtrates and concentrate by heating to evaporate water until the final volume is reduced to 10 mL, yielding a stock solution with a concentration equivalent to 1.0 g of original dried SR material per mL (1.0 g/mL). Aliquot and store at −20 °C. Dilute with low-glucose DMEM complete medium to the desired concentration for cell administration (prepared fresh before use).

#### 2.3.2. Preparation of Stock Solution for Animal Experiments

Accurately weigh 84 g of dried SR, obtain the filtrate using the same decoction method as above, and concentrate by heating to evaporate water until the final volume is reduced to 70 mL, yielding a stock solution with a concentration equivalent to 1.2 g of original dried SR material per mL (1.2 g/mL). Aliquot and store at −20 °C. Dilute with normal saline to the required concentration for animal administration (prepared immediately before use).

The entire extraction, concentration, low-temperature storage, and differentiated solvent dilution protocols for in vivo and in vitro use were adapted from the existing pre-clinical general process for SR aqueous extract, with only the amount of raw material and final concentration of stock solutions adjusted according to the dosage requirements of the cell and animal experiments in this study.

### 2.4. Animal Experimental Design and Grouping

Mate Kunming mice (6–8 weeks old, body weight 18–22 g) were purchased from the Animal Center of Guizhou Medical University and housed in a standard laboratory animal room (temperature 22 ± 2 °C, relative humidity 50 ± 10%, 12 h light/dark cycle) with ad libitum access to food and water. After one week of adaptive feeding, mice were randomly divided into six groups (*n* = 6 per group): Control group, NaAsO_2_ group (10 mg/kg), SR low-dose group (3 g/kg), SR medium-dose group (6 g/kg), SR high-dose group (12 g/kg), and the positive drug sodium 2,3-dimercapto-1-propanesulfonate (DMPS) group (5 mg/kg).

All drugs were prepared with normal saline. To avoid direct gastrointestinal interaction between the test substances, SR and DMPS were administered by gavage at 10:00 am, and NaAsO_2_ was administered 4 h later as a separate gavage. This staggered schedule was intended to minimize the potential for luminal chelation or absorption interference while allowing the herbal extract to achieve systemic distribution prior to the arsenic challenge. Four hours later, all groups except the Control group (which received normal saline) were given NaAsO_2_. The gavage volume was 10 mL/kg. The DMPS group received DMPS for three days followed by a four-day break, and the total modeling period lasted 30 days.

### 2.5. Behavioral Tests

Behavioral tests were conducted before the end of the experiment, including the elevated O-maze and Morris water maze.

Elevated O-maze: Mice were placed in the middle of the open arm of the elevated O-maze in a quiet and dimly lit environment and were allowed to explore freely for 5 min. Movement trajectory and open arm occupancy were recorded using a digital video camera mounted above the apparatus and analyzed with Labmaze V3.0 automated behavioral tracking software (Beijing Zhongshi Dichuang Technology Development Co., Ltd., Beijing, China) to assess anxiety-like behavior.

Morris water maze: The water maze was divided into four quadrants. In a quiet and dimly lit environment, mice underwent water maze escape training for four consecutive days, four trials per day. They were placed into the water from the side wall of each of the four quadrants, with their heads facing the wall. Swim trajectories were recorded using a digital video camera suspended above the pool, and the time taken to reach the platform (escape latency) each day was extracted using Labmaze V3.0 automated behavioral tracking software (Beijing Zhongshi Dichuang Technology Development Co., Ltd., Beijing, China). After the four-day training period, the platform was removed on the fifth day. Mice were placed into the water from the quadrant opposite to the target quadrant, and crossing frequency and target quadrant duration were recorded.

### 2.6. Brain Histopathological Examination

After the behavioral tests, mice were sacrificed, and the brains were removed and sagittally sectioned into three parts. One part was fixed in 4% paraformaldehyde for 24 h. The hippocampus, cerebellum, and cortex were dehydrated, cleared, and paraffin-embedded, then sectioned at 4 μm and stained with hematoxylin and eosin (H&E) after deparaffinization and rehydration. The pathological morphology of neurons in each brain region was observed under a light microscope and photographed.

### 2.7. Cell Culture

The mouse hippocampal neuronal cell line HT22 was purchased from Wuhan Pronocell Biotechnology Co., Ltd. (Wuhan, China) and cultured in low-glucose DMEM supplemented with 10% FBS and 1% penicillin-streptomycin at 37 °C in a 5% CO_2_ atmosphere.

### 2.8. Cell Viability

HT22 hippocampal neurons were seeded in 96-well plates at a density of 8 × 10^4^ cells/well. After 24 h of attachment, the cells were divided into three groups: (1) control, (2) NaAsO_2_ treatment, and (3) NaAsO_2_ + SR intervention.

For the intervention groups, a standard pretreatment and co-treatment protocol was employed, adapted from previously established methodologies [37]. Briefly, cells were pretreated with different concentrations of SR for 4 h to initiate potential intracellular cytoprotective responses prior to the toxic insult. Subsequently, the culture medium was carefully discarded to eliminate any direct extracellular interaction between SR and NaAsO_2_, and replaced with fresh complete medium containing the corresponding concentration of SR and 12.5 μM NaAsO_2_ for a further 24 h of co-treatment. This design was chosen to simulate a clinically relevant scenario of preventive intervention, where the herbal extract is present both before and during the toxic exposure.

At the end of the incubation period, cell viability was assessed using the CCK-8 assay. CCK-8 solution was added to each well and incubated in the dark according to the manufacturer’s instructions. The absorbance was measured at 450 nm using a microplate reader, and the relative cell viability of each group was calculated as a percentage of the control group.

### 2.9. LDH Release Measurement

After 24 h of plating in 96-well plates, cells were treated with drugs. Control and experimental wells (NaAsO_2_ treatment group, NaAsO_2_ + SR intervention group) were set up. The cell supernatant was collected, and LDH release was assessed by measuring the absorbance at 490 nm according to the manufacturer’s instructions for the LDH kit (Nanjing Jiancheng Bioengineering Institute, Nanjing, China).

### 2.10. Scanning Electron Microscopy for Pyroptotic Morphology

Control, NaAsO_2_, and NaAsO_2_ + SR intervention groups were established. Following 24 h of drug exposure, cells were washed with phosphate-buffered saline (PBS) and fixed in 2.5% glutaraldehyde at room temperature, and stored at 4 °C. Samples were washed with pure water, dehydrated through a graded ethanol series, mounted on conductive adhesive, sputter-coated with gold, and observed under a scanning electron microscope to capture ultrastructural images of the cells.

### 2.11. Western Blot Analysis

Brain tissues from each group of mice or drug-treated HT22 cells were lysed in radioimmunoprecipitation assay (RIPA) lysis buffer supplemented with 1 mM phenylmethylsulfonyl fluoride (PMSF) on ice. Protein quantification was carried out using a BCA kit (Nanjing KeyGen Biotech Co. Ltd., Nanjing, China). After lysis on ice for 30 min, samples were centrifuged at 12,000 rpm for 15 min at 4 °C, and the supernatants were collected. Following protein concentration determination by BCA assay, 5× SDS-PAGE loading buffer was added, and the samples were boiled for denaturation. Equal amounts of protein (60 μg total protein) were separated by SDS-PAGE using a 5% stacking gel and 8% or 12% separating gel, according to the method of Laemmli. A prestained protein ladder was used as the molecular weight standard. Proteins were then transferred to PVDF membranes. After washing and blocking, the membranes were incubated overnight at 4 °C with the following primary antibodies: NLRP3 (1:1000), Caspase-1 (1:1000), cleaved Caspase-1 (p20) (1:500), GSDMD (1:1000), GSDMD-N (1:500), IL-1β (1:1000), IL-18 (1:500), and β-actin (1:5000). Following TBST washes, the membranes were incubated with appropriate HRP-conjugated secondary antibodies for 1 h at room temperature, and chemiluminescent signals were visualized using enhanced chemiluminescence (ECL) detection reagent and captured with a fully automated gel imaging system (Tanon 5200, Tanon Science & Technology, Shanghai, China). Densitometric analysis was performed using Tanon AllDoc-X 2.0.0.0 software (Shanghai, China), and the relative expression levels of target proteins were expressed as the ratio of their gray values to that of β-actin.

### 2.12. RNA Isolation and qRT-PCR Analysis

RNA was purified from HT22 cells and brain tissues (treated with drugs) using TRIzol. The samples underwent lysis, chloroform extraction, isopropanol precipitation, ethanol washing, and drying, and were then dissolved in nuclease-free water to obtain purified total RNA. RNA concentration and purity were measured using an ultra-micro spectrophotometer. Only RNA samples with A260/A280 ratios between 1.8 and 2.0 were used for subsequent experiments.

Two micrograms of qualified RNA were reverse transcribed into cDNA according to the kit instructions on a conventional thermal cycler (Bio-Rad, Hercules, CA, USA). A 10 μL qPCR reaction system was prepared using SYBR Green dye, and relative mRNA expression of target genes was determined on a real-time PCR detection system (Bio-Rad, Hercules, CA, USA). The target genes analyzed were NLRP3, Caspase-1, GSDMD, IL-1β, and IL-18, with β-actin used as the internal reference gene. Relative gene expression was calculated using the 2^−ΔΔCt^ method.

The primer sequences used in this study are listed in Table S1 (Supplementary Material).

### 2.13. Statistical Analysis

Statistical analyses and graphing were performed using GraphPad Prism (Version 10.4.1, GraphPad Software, San Diego, CA, USA). Data are presented as mean ± SD. For two-group comparisons, an unpaired two-tailed Student’s *t*-test was used. For multi-group comparisons, ANOVA followed by Tukey’s post hoc test was employed. *p* < 0.05 was considered statistically significant. Complete ANOVA results, including F values and degrees of freedom for all significant comparisons, are provided in Supplementary Table S2.

## 3. Results

### 3.1. Saposhnikovia Radix Aqueous Extract Ameliorates Behavioral and Cognitive Impairments Induced by NaAsO_2_ in Mice

To evaluate the effects of SR aqueous extract on the emotional state and cognitive function of NaAsO_2_-exposed mice, we conducted behavioral assessments using the elevated O-maze and Morris water maze.

**Figure 1:**
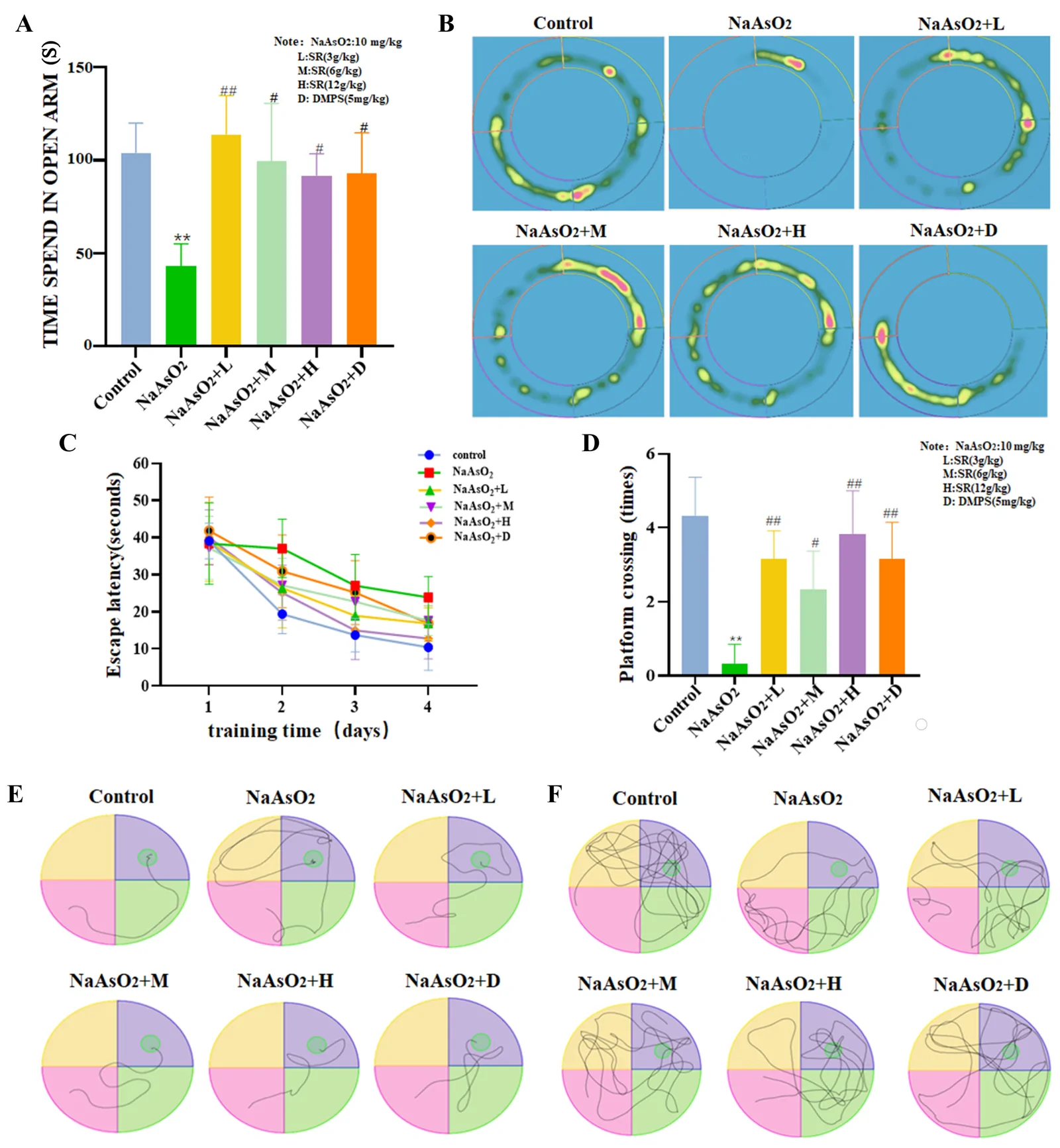
Saposhnikoviae Radix aqueous extract ameliorates depressive-like behavior and learning/memory impairment in NaAsO_2_-exposed mice. (**A**) Open-arm residence time in the elevated O-maze; (**B**) thermal imaging trajectories in the O-maze. The colors green, yellow, and red correspond to mouse presence frequency in ascending order (low to high); (**C**) changes in escape latency over time; (**D**) number of original platform crossings; (**E**) representative path tracings on day 4 of training; (**F**) representative path tracings during the memory test. Black lines in (**E**,**F**) indicate the swimming paths of mice in the maze. Values (mean ± SD) are shown; ** *p* < 0.01 vs. Control; # *p* < 0.05, ## *p* < 0.01 vs. NaAsO_2_ model, *n* = 6.

The O-maze results showed that the time spent in the open arms reflects the anxiety level of mice (Figure 1A). Compared with the Control group, the open-arm residence time in the NaAsO_2_ model group was significantly reduced (*p* < 0.01), indicating that NaAsO_2_ exposure induced pronounced anxiety-like behavior in mice. Compared with the NaAsO_2_ group, the open-arm time was increased significantly in all tested doses of SR and DMPS significantly attenuated NaAsO_2_-induced compared to the model group (*p* < 0.05, *p* < 0.01). Among them, the medium-dose and high-dose groups exhibited more prominent improvements, comparable to that of the DMPS group. While low dose of SR appears to give a higher response, statistical analysis revealed no significant differences among the low, medium, and high dose groups. The trajectory heatmaps (Figure 1B) visually showed that the activity of mice in the NaAsO_2_ model group was highly concentrated in the closed arms, whereas after administration of SR or the positive drug, the activity range and frequency of mice in the open arms were markedly increased.

The Morris water maze place navigation test results (Figure 1C) showed that the escape latencies of all groups decreased with training days over the 4-day training period. At the same training time points, there was a significant increase in escape latency in the NaAsO_2_ group compared with the control, suggesting that NaAsO_2_ exposure impaired the spatial learning ability of mice. Following graded doses of SR or positive drug intervention, the daily escape latencies of each group were significantly shortened compared with the NaAsO_2_ model group, and the improvement showed an increasing trend with higher doses. On the 4th day of training, the swimming paths of mice during the escape latency test were recorded (Figure 1E). The movement trajectory of the NaAsO_2_ model group was disorganized, rarely remaining in the target quadrant where the platform was originally located, whereas the trajectories of the SR-treated groups and the positive drug DMPS group were concentrated in the target quadrant, further supporting that the tested drug can restore impaired spatial memory. The spatial probe test evaluated long-term spatial memory function by the number of platform crossings. On day 5 (probe trial), the platform was removed, and mice were released from the opposite quadrant. Platform crossings and swimming distance in the target quadrant are presented in Figure 1D,F. The control group showed the greatest number of platform crossings, whereas a marked reduction was observed in the NaAsO_2_ model group. (*p* < 0.01), confirming that NaAsO_2_ caused severe spatial memory deficits. Compared with the NaAsO_2_ model group, the number of platform crossings was significantly increased in the NaAsO_2_ + L, NaAsO_2_ + M, NaAsO_2_ + H, and DMPS groups (*p* < 0.05). The memory-restorative effects of the medium- and high-dose groups did not differ significantly from those of the DMPS group. Taken together, these behavioral results indicate that NaAsO_2_ exposure simultaneously causes anxiety-like behavior and dual impairments in spatial learning and memory in mice, and the model is stable and reliable. Low-, medium-, and high-dose SR administration can alleviate these behavioral abnormalities, with the medium and high doses displaying overall intervention effects comparable to that of DMPS.

### 3.2. Saposhnikovia Radix Aqueous Extract Attenuates Brain Histopathological Damage in NaAsO_2_-Exposed Mice

**Figure 2:**
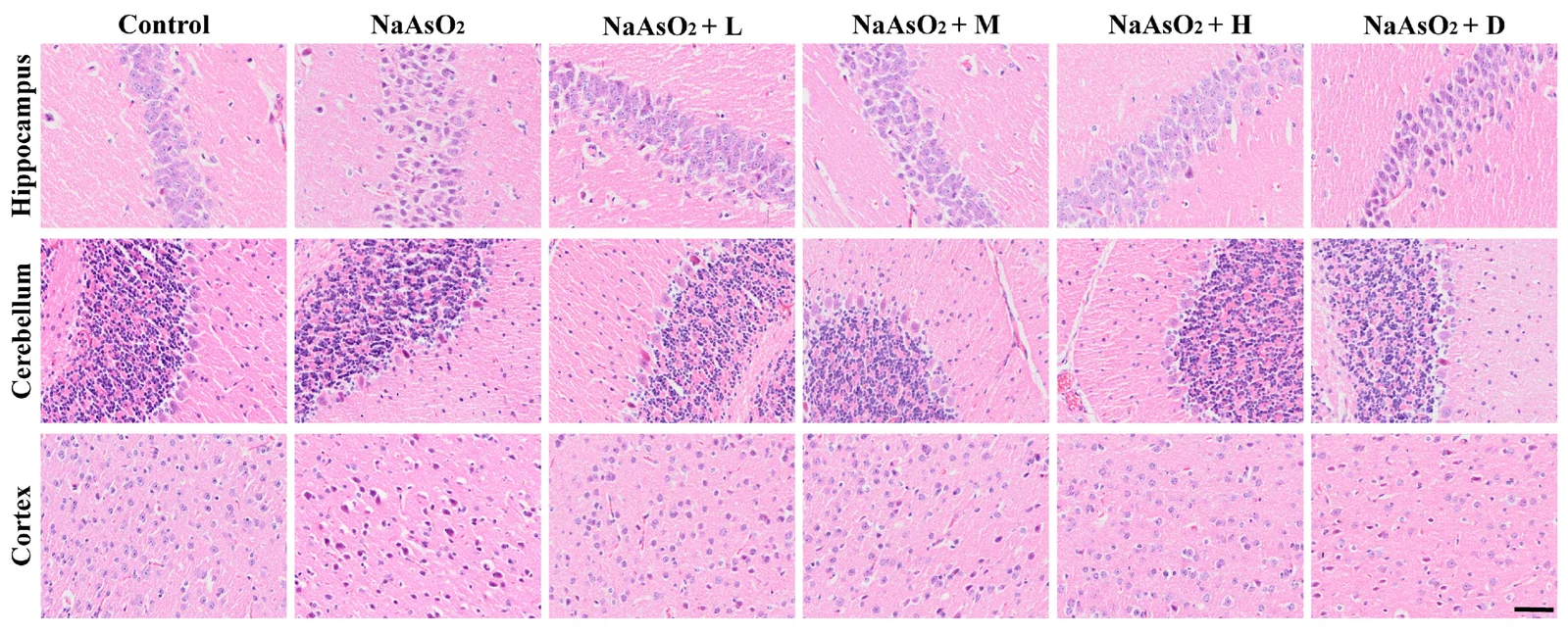
Saposhnikovia Radix aqueous extract attenuates brain histopathological damage in NaAsO_2_-exposed mice (*n* = 6), Scale bar = 60 μm.

H&E staining confirmed the presence of intact neuronal architecture, regular arrangement, uniform nuclear staining, and no obvious cellular shrinkage, vacuolar degeneration, or inflammatory infiltration in the hippocampus, cerebellum, and cerebral cortex of the Control group (Figure 2). The brain tissue of the NaAsO_2_ group exhibited significant pathological damage: in the hippocampus, pyramidal cells were loosely and irregularly arranged, with numerous shrunken cell bodies, deeply stained pyknotic nuclei, and widened intercellular spaces; in the cerebellum, the density of granule cells was decreased, Purkinje cells were reduced in number and showed atrophic morphology; in the cerebral cortex, extensive neuronal vacuolar degeneration and nuclear fragmentation were observed, with obvious histopathological damage, confirming that NaAsO_2_ exposure caused neuronal injury in multiple brain regions of mice. After intervention with low-, medium-, and high-dose SR (NaAsO_2_ + L, NaAsO_2_ + M, NaAsO_2_ + H) and the DMPS, the pathological damage in the hippocampus, cerebellum, and cerebral cortex was alleviated to varying degrees. The improvement was more significant in the medium- and high-dose groups (NaAsO_2_ + M and NaAsO_2_ + H): neuronal arrangement tended to be orderly, the number of cells with pyknotic nuclei and vacuolar degeneration was greatly reduced, and the brain tissue morphology approached that of the Control group, with a protective effect comparable to that of the DMPS group. The low-dose NaAsO_2_ + L only mildly reduced the brain histopathological damage. These pathological results indicate that SR can dose-dependently ameliorate NaAsO_2_-induced neuronal damage in the hippocampus, cerebellum, and cerebral cortex, demonstrating a clear protective effect on the central nervous system (Figure 2).

### 3.3. Saposhnikovia Radix Aqueous Extract Suppresses Pyroptosis and Inflammation Activation in the Brain of NaAsO_2_-Exposed Mice

**Figure 3:**
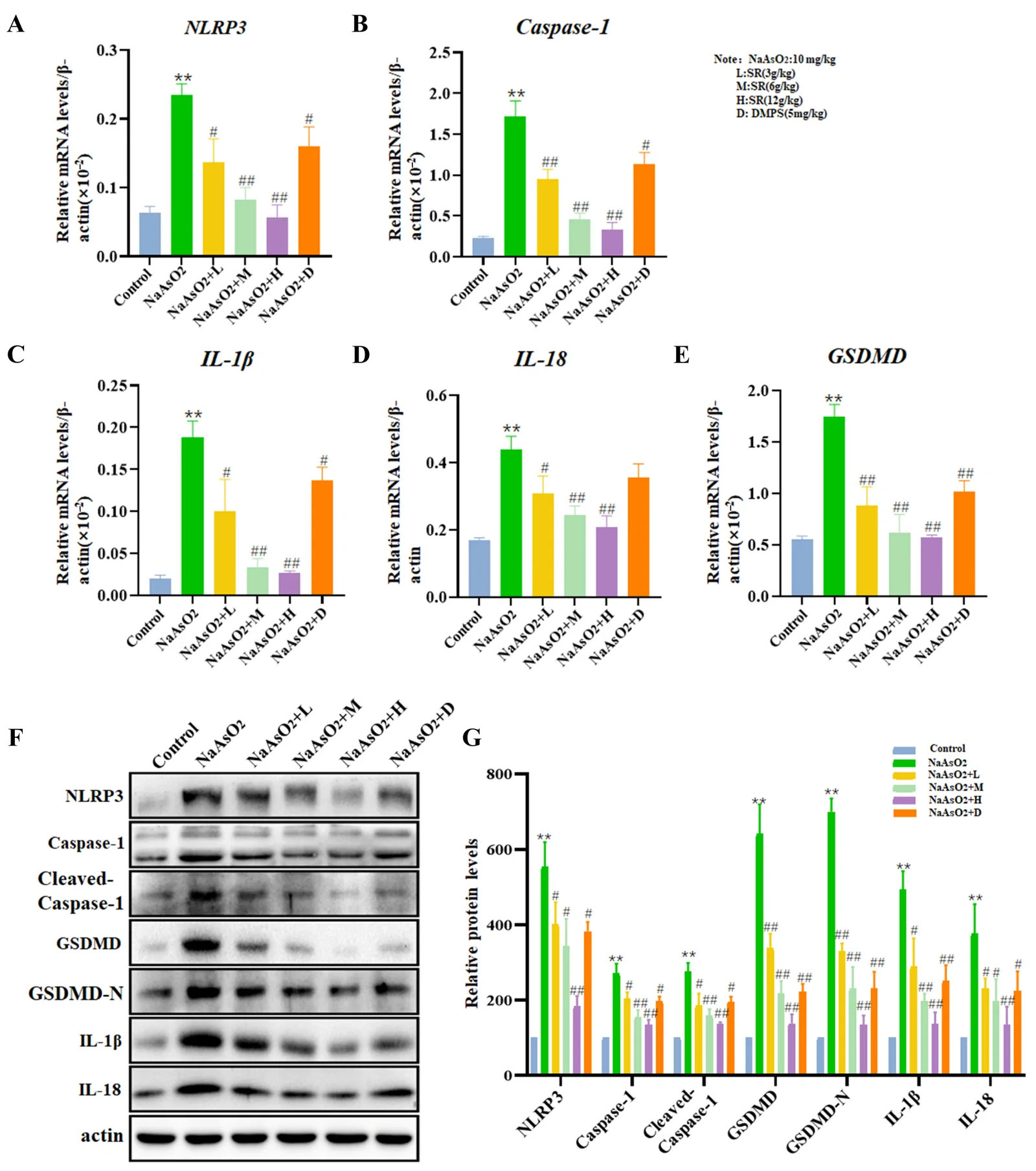
Saposhnikovia Radix aqueous extract inhibits the activation of the NLRP3/Caspase-1/GSDMD pyroptosis pathway in mouse brain. (**A**–**E**) qRT-PCR quantification of the expression of NLRP3, Caspase-1, IL-18, IL-1β and GSDMD mRNAs in the brain tissue of each group; (**F**) representative immunoblots for NLRP3, caspase-1 (total and cleaved), GSDMD (full-length and N-terminal fragment), IL-18, IL-1β and β-actin loading control in the brain tissue of each group; (**G**) quantitative statistical results of protein band gray values corresponding to panel (**F**), with relative protein expression levels normalized to β-actin. Group annotations: Control, NaAsO_2_, NaAsO_2_ + L, NaAsO_2_ + M, NaAsO_2_ + H, model groups receiving low-, medium-, and high-dose test substance intervention, respectively; NaAsO_2_ + D, positive drug group. Values (mean ± SD) are shown; ** *p* < 0.01 vs. Control; ## *p* < 0.01, # *p* < 0.05 vs. NaAsO_2_ model.

qRT-PCR results (Figure 3A–E) showed that compared with the Control group, the relative mRNA expression levels of NLRP3, Caspase-1, IL-1β, IL-18, and GSDMD in the brain tissue of the NaAsO_2_ model group were all significantly increased (*p* < 0.01), indicating that NaAsO_2_ exposure significantly activated the NLRP3 inflammasome and the transcription of downstream pyroptosis-related genes. Compared with the NaAsO_2_ model group, the mRNA expression of these genes was downregulated to varying degrees in the NaAsO_2_ + L, NaAsO_2_ + M, NaAsO_2_ + H, and DMPS groups (*p* < 0.05, *p* < 0.01), and the inhibitory effect gradually increased with increasing dose, with the high-dose NaAsO_2_ + H group showing a magnitude of gene transcription downregulation similar to that of DMPS group.

Western blot protein band and quantitative statistical results (Figure 3F,G) showed a consistent trend with gene transcription levels: the relative protein levels of NLRP3, Caspase-1, activated Cleaved-Caspase-1, full-length GSDMD, pyroptosis-executing fragment GSDMD-N, and mature pro-inflammatory cytokines IL-1β and IL-18 in the NaAsO_2_ model group were significantly increased compared with the normal Control group (*p* < 0.01). After intervention with low-, medium-, and high-dose SR, the expression levels of key pathway proteins were significantly reduced compared with the NaAsO_2_ model group (*p* < 0.05, *p* < 0.01). The medium- and high-dose groups showed more prominent inhibition of the activated pyroptosis proteins Cleaved-Caspase-1 and GSDMD-N and the pro-inflammatory factors IL-1β and IL-18, with an overall regulatory effect comparable to that of the positive control drug.

Combined gene and protein quantification results confirm that NaAsO_2_ exposure can hyperactivate the NLRP3 inflammasome signaling axis in brain tissue, initiating Caspase-1-dependent GSDMD-mediated pyroptosis and releasing high concentrations of IL-18 and IL-1β. Graded doses of SR can dose-dependently inhibit the gene and protein activation of the NLRP3/Caspase-1/GSDMD pyroptosis pathway, reduce the release of inflammatory factors, and thereby alleviate NaAsO_2_-induced central nervous system inflammation and neuronal pyroptotic injury.

### 3.4. Saposhnikovia Radix Aqueous Extract Alleviates Pyroptotic Morphological Damage in NaAsO_2_-Exposed HT22 Cells

**Figure 4:**
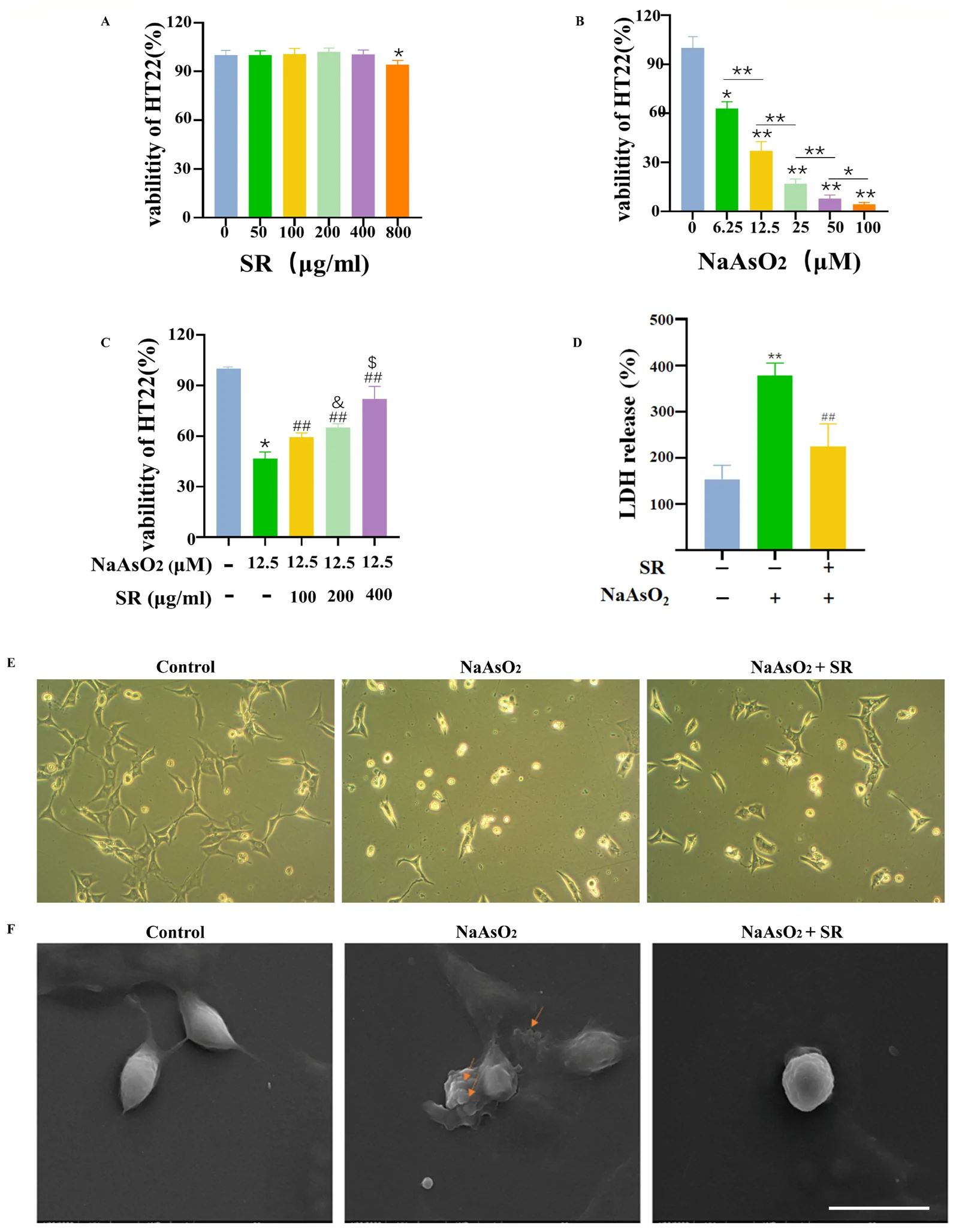
Saposhnikovia Radix aqueous extract alleviates NaAsO_2_-induced neuronal cytotoxicity and pyroptotic morphological damage. (**A**) Effect of SR on HT22 cell viability; (**B**) effect of NaAsO_2_ on HT22 cell viability; (**C**) effect of SR and NaAsO_2_ on HT22 cell viability; for panels (**A**–**C**), cell viability was assessed by CCK-8 assay and is expressed as a percentage of the untreated control group (% of control). (**D**) Effect of SR and NaAsO_2_ on LDH release in HT22 cells; LDH release is expressed as a percentage of maximum LDH release (% of max). (**E**) Effect of SR and NaAsO_2_ on HT22 cell morphology; (**F**) effect of SR and NaAsO_2_ on pyroptotic body formation in HT22 cells; red arrows indicate pyroptotic bodies. scale bar = 20 μm. For panels (**D**–**F**), cells were treated with 12.5 μM NaAsO_2_. Values (mean ± SD) are shown; * *p* < 0.05 and ** *p* < 0.01 vs. control; ## *p* < 0.01 vs. NaAsO_2_; & *p* < 0.05 vs. NaAsO_2_ + 100 μg/mL SR; $ *p* < 0.05 vs. NaAsO_2_ + 200 μg/mL SR.

The CCK-8 cell viability assay (Figure 4A) showed that after treatment with gradient concentrations of SR alone (50–400 μg/mL), cell viability was not statistically different from the blank control group. Only the high concentration of 800 μg/mL slightly reduced cell viability (*p* < 0.05), suggesting that 400 μg/mL is a safe concentration of SR with no obvious cytotoxicity, and can be used for subsequent cell intervention experiments. The gradient NaAsO_2_ toxicity assay (Figure 4B) demonstrated that with increasing NaAsO_2_ concentration, HT22 cell viability showed a significant concentration-dependent decrease. In comparison with the control, 6.25 μM NaAsO_2_ already significantly inhibited cell survival (*p* < 0.05), and 25, 50, and 100 μM NaAsO_2_ treatment caused highly significant decreases in cell viability (*p* < 0.01). Therefore, 12.5 μM NaAsO_2_ was selected to construct the in vitro neuronal injury model for subsequent protection experiments. The cell co-treatment viability results (Figure 4C) showed that compared with the control, the cell viability of the NaAsO_2_ model group was significantly reduced (*p* < 0.05). Compared with the NaAsO_2_ model group, 100, 200, and 400 μg/mL SR intervention all significantly restored cell viability (*p* < 0.01), and the protective effect gradually increased with increasing SR concentration. The 400 μg/mL high-dose group exhibited the best improvement, significantly superior to the 100 and 200 μg/mL low- and medium-dose groups (*p* < 0.05, *p* < 0.05), displaying an obvious dose-dependent neuroprotective characteristic. Compared with the control, the NaAsO_2_ model group exhibited a highly significant increase in supernatant LDH release (Figure 4D) reflecting cell membrane damage (*p* < 0.01). After SR intervention, LDH release levels were significantly reduced (*p* < 0.01), indicating that SR can alleviate NaAsO_2_-induced cell membrane leakage damage. Conventional light microscopy (Figure 4E) revealed that untreated HT22 cells retained normal morphology, characterized by plump cell bodies, extensive neuritic projections, and intact intercellular connections. Conversely, exposure to NaAsO2 induced pronounced cytomorphological alterations, including diminished cell survival, somal atrophy, widespread neuritic fragmentation, and elevated numbers of non-adherent cells. Intriguingly, the addition of SR effectively rescued these pathological changes, as reflected by an increased number of viable cells and a notable recovery of neurite organization. Scanning electron microscopy of the cell membrane ultrastructure (Figure 4F) showed that the cell membranes of the control group were smooth, continuous, and structurally intact. In the NaAsO_2_ model group, numerous bulging bubbles and damaged pores appeared on the cell membrane surface, displaying typical pyroptotic morphological features (marked by red arrows). After SR intervention, the membrane blebbing and damage were significantly reduced, and the membrane structure tended toward integrity. The above in vitro experimental results confirm that NaAsO_2_ can dose-dependently induce a decline in HT22 hippocampal neuron viability, rupture the cell membrane, and trigger pyroptosis-like injury. Within a non-toxic concentration range, SR can dose-dependently reverse the NaAsO_2_-induced decrease in cell viability, reduce LDH leakage, and improve overall cell morphology and cell membrane ultrastructure, exerting a direct in vitro protective effect against NaAsO_2_-induced pyroptotic injury in hippocampal neurons.

### 3.5. Saposhnikovia Radix Aqueous Extract Inhibits Pyroptosis and Inflammatory Responses in NaAsO_2_-Exposed HT22 Cells

**Figure 5:**
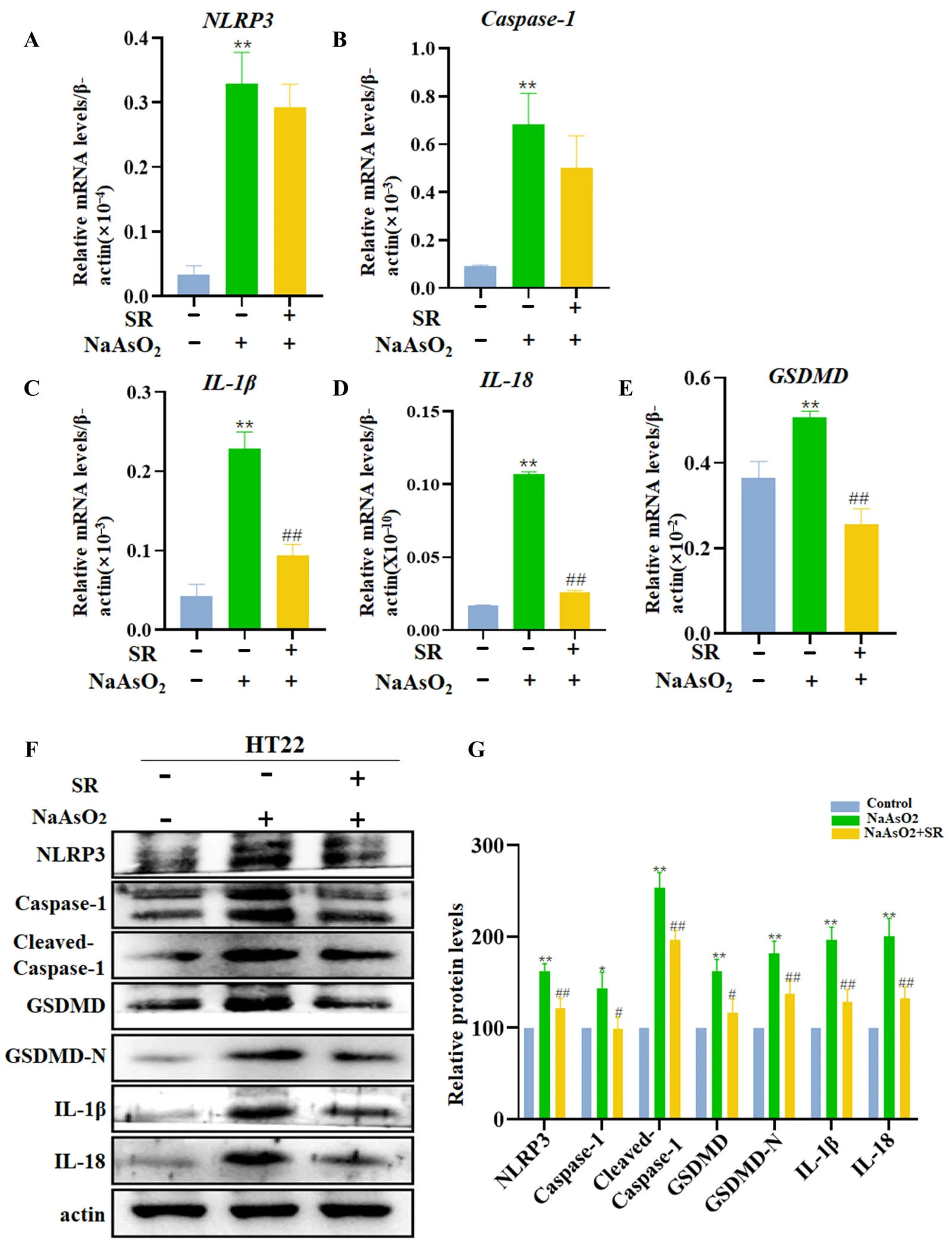
Saposhnikovia Radix aqueous extract affects mRNA and protein expression of pyroptosis-associated factors in NaAsO_2_-exposed HT22 cells. (**A**–**E**) qRT-PCR detection of pyroptosis-related gene mRNA expression; (**F**) representative Western blot bands of pyroptosis-related proteins; (**G**) quantitative statistical analysis of protein band gray values. For all panels, cells were treated with NaAsO_2_ (12.5 μM) in the presence or absence of SR (400 μg/mL). Values (mean ± SD) are shown; * *p* < 0.05 and ** *p* < 0.01 vs. Control; # *p* < 0.05 and ## *p* < 0.01 vs. NaAsO_2_.

To clarify the regulatory effect of SR aqueous extract on the pyroptosis pathway, we conducted qRT-PCR and Western blot analyses at both the transcriptional and protein levels. qRT-PCR results (Figure 5A–E) showed that compared with the blank control group, the relative mRNA expression levels of NLRP3, Caspase-1, IL-1β, IL-18, and GSDMD in the NaAsO_2_-treated model group were all significantly increased (*p* < 0.01), indicating that NaAsO_2_ exposure significantly activated NLRP3 inflammasomes and the transcription of downstream pyroptosis-related genes in HT22 hippocampal neurons. Compared with the model group, the mRNA expression levels of IL-1β, IL-18, and GSDMD in the SR intervention group were significantly downregulated (*p* < 0.01). The mRNA levels of NLRP3 and Caspase-1 also showed a decreasing trend, but these changes did not reach statistical significance (Figure 5A–E), demonstrating that SR can effectively suppress the excessive transcription of pyroptosis pathway genes induced by NaAsO_2_.

Western blot protein band and quantitative statistical results (Figure 5F,G) showed a trend consistent with the transcriptional level: compared with the control group, the relative protein expression levels of NLRP3, Caspase-1, activated Cleaved-Caspase-1, full-length GSDMD, pyroptosis-executing fragment GSDMD-N, and mature pro-inflammatory cytokines IL-1β and IL-18 in the NaAsO_2_ model group were all highly significantly increased (*p* < 0.01). After SR intervention, the expression levels of key pathway proteins were significantly reduced compared with the NaAsO_2_ model group (*p* < 0.05, *p* < 0.01), with the downregulation of activated pyroptosis markers and pro-inflammatory factors such as Cleaved-Caspase-1, GSDMD-N, IL-1β, and IL-18 being particularly pronounced.

Combined transcriptional and protein quantification results at the cellular level confirm that NaAsO_2_ exposure can hyperactivate the NLRP3/Caspase-1/GSDMD signaling axis in HT22 neurons, initiating Caspase-1-dependent GSDMD-mediated pyroptosis and strongly increasing the levels of IL-18 and IL-1β. SR can simultaneously suppress the abnormal activation of this pyroptosis-inflammatory pathway at both the gene transcription and protein translation levels, thereby alleviating NaAsO_2_-induced pyroptotic injury and inflammatory response in hippocampal neurons.

## 4. Discussion

The anxiety-like behavior and spatial cognitive impairment induced by arsenic exposure represent functional readouts of organic damage to central neurons—a stable and reproducible toxic phenotype that has been corroborated across multiple independent rodent models, providing a reliable basis for pharmacodynamic evaluation within the neuroexposome framework [12,13,15,16,47,48]. Using this established model, we compared the intervention effects of SR aqueous extract with those of the classical clinical antidote DMPS. Notably, both agents effectively ameliorated arsenic-induced neurological deficits; however, their mechanisms appear to differ. DMPS functions primarily through systemic chelation of free arsenic ions, promoting toxicant metabolism and excretion—an approach that blocks ongoing exposure at the source but cannot reverse established central neuroinflammation or programmed neuronal death [15,49,50,51]. It should be noted that DMPS was used as a positive control exclusively in the in vivo experiments to validate systemic arsenic detoxification. In the in vitro studies, which focused on the direct anti-pyroptotic mechanisms of SR, DMPS was not included, as its membrane-impermeable chelating action has limited pharmacological relevance in a closed cell culture system.

In contrast, the in vitro data suggest that SR aqueous extract may exert neuroprotection, at least in part, through indirect modulation of the pyroptosis-inflammatory axis, independent of systemic toxicant removal. This mechanistic finding is fundamentally distinct from our previous report on the PI3K/AKT/ferroptosis pathway [36], which was an exclusively in vitro, network pharmacology-driven investigation. The present study employs a phenotype-driven approach with integrated in vivo behavioral, histopathological, and ultrastructural analyses, providing a novel translational framework for understanding and counteracting arsenic neurotoxicity.

Histopathological analysis revealed that arsenic induced pathological alterations across multiple brain regions, including the hippocampus, cerebral cortex, and cerebellum, and SR administration broadly ameliorated these alterations, preserving normal neuronal morphology. This pattern of damage is consistent with the known non-selective central toxicity of arsenic [8,9,11,12,14,15,28]. The regional neuroprotection observed at the histological level provides a structural correlate for the concurrent improvement in both emotional (anxiety-related) and cognitive (spatial memory) domains observed in our behavioral assays.

Interestingly, we observed that SR significantly reduced NLRP3 and Caspase-1 at the protein level, while their mRNA expression was only modestly and non-significantly decreased. This transcriptional–translational dissociation suggests that SR may regulate NLRP3 and Caspase-1 primarily through post-transcriptional mechanisms—such as enhanced protein degradation or inhibition of translation—rather than through transcriptional suppression. This finding aligns with the emerging understanding that NLRP3 inflammasome activation is predominantly controlled at the post-translational level, and highlights a potential mechanism by which SR exerts its anti-pyroptotic effects. This mechanistic feature implies that SR may not merely dampen the inflammatory response globally, but could specifically interfere with the execution phase of pyroptosis—a more targeted intervention that might preserve beneficial immune surveillance functions while curtailing pathological inflammation.

The in vitro HT22 experiments further corroborated this interpretation. By completely eliminating systemic confounding factors, the single-cell-type system confirmed that SR can directly target hippocampal neurons, inhibiting arsenic-induced pyroptosis activation and inflammatory responses in vitro. The consistency between in vivo and in vitro findings, both showing protein-level suppression of the pyroptotic cascade, supports the interpretation that SR’s neuroprotection involves a direct pharmacological action on the neuronal pyroptosis pathway, which is at least partially independent of systemic toxicant clearance. Future studies incorporating measurements of arsenic concentrations in target tissues with and without SR pretreatment will be necessary to dissect the relative contributions of pharmacodynamic and pharmacokinetic mechanisms to the observed neuroprotection.

Importantly, our study extends beyond conventional antioxidant and non-specific anti-inflammatory frameworks that have dominated natural product research in arsenic neurotoxicity [31,35,52,53]. By focusing specifically on the NLRP3/Caspase-1/GSDMD pyroptotic axis, we have identified a novel protective mechanism of SR—one that may intercept the exposure-to-pathology cascade at its pyroptotic execution phase, potentially uncoupling environmental arsenic exposure from its neuropathological consequences. This finding suggests that SR warrants further investigation as a pathway-modulating agent within the neuro-exposome paradigm.

Nevertheless, several limitations warrant acknowledgment. First, although we have identified post-transcriptional regulation as the likely mechanism underlying the mRNA-protein dissociation, the precise molecular machinery—whether ubiquitin-proteasome degradation, autophagy-lysosomal pathway, or translational inhibition—remains to be defined. Second, SR aqueous extract is a complex mixture; the specific bioactive components responsible for the observed pyroptosis inhibition have not been identified. Third, while our in vitro data demonstrate direct neuronal targeting, we have not performed target gene validation (e.g., NLRP3 or GSDMD knock-out/knockdown) to establish causality definitively. Fourth, arsenic concentrations were not measured in blood or target tissues. However, the well-established pharmacokinetic profile of this dosing regimen [54] and the robust behavioral, histopathological, and molecular phenotypes observed in the NaAsO_2_-exposed groups support the validity of the core mechanistic conclusions. Future studies incorporating arsenic speciation and tissue distribution analyses will be necessary to further strengthen the evidence. Active component screening, pathway-specific inhibitor/activator experiments, and genetic validation should be prioritized in future studies to refine the mechanistic model and accelerate the translational potential of SR-derived therapeutics for arsenic-related neurological disorders.

## 5. Conclusions

Arsenic exposure engages the NLRP3/Caspase-1/GSDMD pyroptotic cascade as a primary molecular translator that converts an environmental insult into sustained neuropathological sequelae, driving synchronous damage across the hippocampus, cerebral cortex, and cerebellum, and culminating in anxiety-like behaviors and spatial cognitive deficits. By demonstrating that SR aqueous extract intercepts this exposure-to-disease trajectory at multiple nodes—blunting NLRP3 inflammasome assembly, suppressing GSDMD-mediated membrane pore formation, and curtailing the downstream IL-1β/IL-18 inflammatory burst—our study establishes that pharmacological recalibration of this pyroptosis-inflammatory axis is both achievable and therapeutically relevant. Notably, at medium and high doses, SR’s neuroprotective efficacy paralleled that of the clinical chelator DMPS, yet through a fundamentally distinct pathway-resetting mechanism rather than toxicant sequestration. Collectively, these findings embed arsenic neurotoxicity within the neuroexposome paradigm by identifying a specific exposure-to-pathology translator, and position SR aqueous extract as a multi-target botanical prototype that rebalances exposure-driven neurophysiological misalignment, providing a mechanistic foundation for integrating traditional medicine into environmental neurotoxicology and offering a promising direction for the prevention of metalloid-induced neurological disorders.

## Data Availability

The original contributions presented in this study are included in the article/Supplementary Material. Further inquiries can be directed to the corresponding authors.

## Supplementary Materials

The following supporting information can be downloaded at: https://www.mdpi.com/article/10.3390/toxics14080706/s1, Table S1: Primer sequence; Table S2: Summary of one-way ANOVA results for all significant comparisons.
