# Supplementary material for: Aqueous Extract of Saposhnikoviae Radix Antagonizes Arsenic Neurotoxicity via the NLRP3/Caspase-1/GSDMD Pyroptotic Axis: Translating Environmental Exposure into Neuropathology and Herbal Recalibration
Source: Toxics. 2026 Aug 10;14(8):706. doi: 10.3390/toxics14080706 (PMC13517699; doi:10.3390/toxics14080706)
Supplement: Supplementary file 1 [file toxics-14-00706-s001.zip › toxics-4434881-supplementary.pdf]

Supplementary Material

Supplementary Tables

Table S1. Primer sequence.

Gene name		Primer sequence
Caspase-1	Forward Primer	GCCCTCATTATCTGCAACAC
	Reverse Primer	AGCAACTTCATTTCTCTGAGG
GSDMD	Forward Primer	ACCTGGGTCTTGCTAGAAG
	Reverse Primer	TGACAATAGGAACAGGGAGG
NLRP3	Forward Primer	GATCTCGCATTGGTTCTGAG
	Reverse Primer	GGGTCCTTCATCTTTTCACAC
IL-1β	Forward Primer	CCAGCTTCAAATCTCGCAG
	Reverse Primer	ACGGGAAAGACACAGGTAG
IL-18	Forward Primer	TCGTTGACAAAAGACAGCC
	Reverse Primer	GCCAGTCCTCTTACTTCACT
β-actin	Forward Primer	GCTGTGCTATGTTGCTCTAG
	Reverse Primer	CGCTCGTTGCCAATAGTG
TNF-α	Forward Primer	TGTACCTTGTCTACTCCCAG
	Reverse Primer	GGTTGACTTTCTCCTGGTATG

Table S2. Summary of one-way ANOVA results for all significant comparisons.

Figure	Parameter	F (DFn, DFd)	P value
Fig 1A	Open-arm residence time in the levated O-maze	$F(5, 30) = 4.209$	$P=0.0051$
Fig 1D	Number of original platform crossings	$F(5, 25) = 4.954$	$P=0.0024$
Fig 3A	NLRP3 mRNA level	$F(5, 12) = 28.73$	$P<0.0001$
Fig 3B	Caspase-1 mRNA level	$F(5, 12) = 68.89$	$P<0.0001$
Fig 3C	IL-1β mRNA level	$F(5, 12) = 39.86$	$P<0.0001$
Fig 3D	IL18 mRNA level	$F(5, 12) = 22.38$	$P<0.0001$
Fig 3E	GSDMD mRNA level	$F(5, 12) = 38.95$	$P<0.0001$
	NLRP3 protein level	$F(5, 12) = 33.39$	$P<0.0001$
	Caspase-1 protein level	$F(5, 12) = 39.06$	$P<0.0001$
	GSDMD protein level	$F(5, 12) = 105.8$	$P<0.0001$
Fig 3G	IL-1β protein level	$F(5, 12) = 32.22$	$P<0.0001$
	IL18 protein level	$F(5, 12) = 11.22$	$P<0.0001$
Fig 4A	Effect of SR on HT22 cell viability	$F(5, 12) = 3.370$	$P=0.0393$
Fig 4B	Effect of $NaAsO_2$ on HT22 cell viability	$F(5, 12) = 4291$	$P<0.0001$
Fig 4C	Effect of SR and $NaAsO_2$ on HT22 cell viability	$F(4, 10) = 76.80$	$P<0.0001$
Fig 4D	Effect of SR and $NaAsO_2$ on LDH release	$F(2, 6) = 30.16$	$P<0.0001$
